# Author Correction: Meta-analysis of sub-Saharan African studies provides insights into genetic architecture of lipid traits

**DOI:** 10.1038/s41467-022-32072-y

**Published:** 2022-08-02

**Authors:** Ananyo Choudhury, Jean-Tristan Brandenburg, Tinashe Chikowore, Dhriti Sengupta, Palwende Romuald Boua, Nigel J. Crowther, Godfred Agongo, Gershim Asiki, F. Xavier Gómez-Olivé, Isaac Kisiangani, Eric Maimela, Matshane Masemola-Maphutha, Lisa K. Micklesfield, Engelbert A. Nonterah, Shane A. Norris, Hermann Sorgho, Halidou Tinto, Stephen Tollman, Sarah E. Graham, Cristen J. Willer, AWI-Gen study, H3Africa Consortium, Scott Hazelhurst, Michèle Ramsay

**Affiliations:** 1 Sydney Brenner Institute for Molecular Bioscience, Faculty of Health Sciences, University of the Witwatersrand, Johannesburg, South Africa; 2 South African Medical Research Council/University of the Witwatersrand Developmental Pathways for Health Research Unit, Department of Paediatrics, School of Clinical Medicine, Faculty of Health Sciences, University of the Witwatersrand, Johannesburg, South Africa; 3 Clinical Research Unit of Nanoro, Institut de Recherche en Sciences de la Santè, Nanoro, Burkina Faso; 4 Department of Chemical Pathology, National Health Laboratory Service, Faculty of Health Sciences, University of the Witwatersrand, Johannesburg, South Africa; 5 Navrongo Health Research Centre, Ghana Health Service, Navrongo, Ghana; 6 C.K. Tedam University of Technology and Applied Sciences, Navrongo, Ghana; 7 African Population and Health Research Center, Nairobi, Kenya; 8 MRC/Wits Rural Public Health and Health Transitions Research Unit (Agincourt), School of Public Health, Faculty of Health Sciences, University of the Witwatersrand, Johannesburg, South Africa; 9 Department of Public Health, School of Health Care Sciences, Faculty of Health Sciences, University of Limpopo, Polokwane, South Africa; 10 Department of Pathology and Medical Sciences, School of Health Care Sciences, Faculty of Health Sciences, University of Limpopo, Polokwane, South Africa; 11 Department of Internal Medicine, Division of Cardiology, University of Michigan, Ann Arbor, MI 48109 USA; 12 Department of Computational Medicine and Bioinformatics, University of Michigan, Ann Arbor, MI 48109 USA; 13 Department of Human Genetics, University of Michigan, Ann Arbor, MI 48019 USA; 14 School of Electrical and Information Engineering, University of the Witwatersrand, Johannesburg, South Africa; 15 Division of Human Genetics, National Health Laboratory Service and School of Pathology, Faculty of Health Sciences, University of the Witwatersrand, Johannesburg, South Africa

**Keywords:** Genome-wide association studies, Quantitative trait, Cardiovascular diseases, Risk factors

Correction to: *Nature Communications* 10.1038/s41467-022-30098-w, published online 11 May 2022

“The original version of this Article contained an error in Fig. 5, in which the figure was inadvertently replaced with an earlier version containing incorrect AFG values. The correct version of Fig. 5 is:
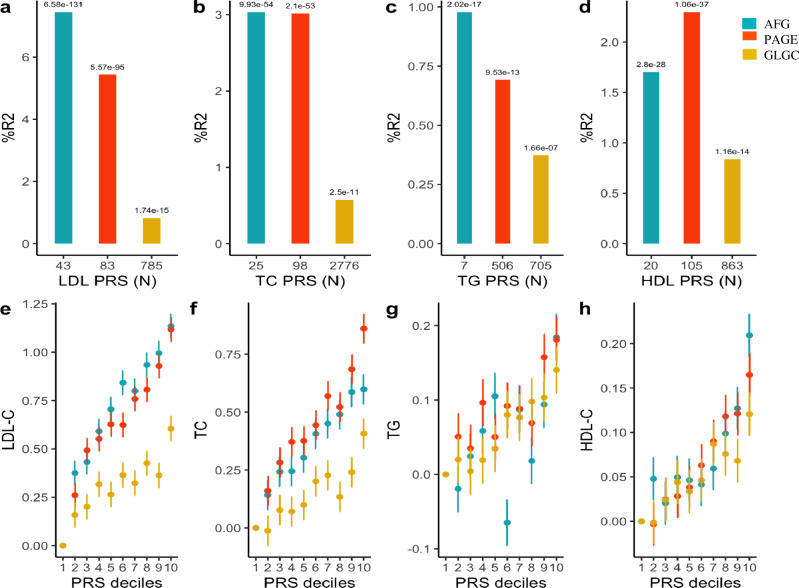


which replaces the previous incorrect version:
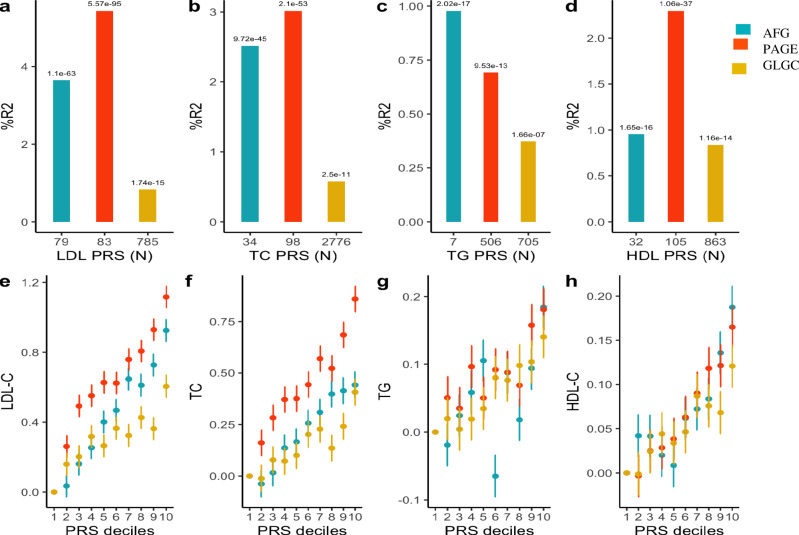


This has been corrected in both the PDF and HTML versions of the Article.”

