# Supplementary material for: Meta-analysis of sub-Saharan African studies provides insights into genetic architecture of lipid traits
Source: Nat Commun. 2022 May 11;13:2578. doi: 10.1038/s41467-022-30098-w (PMC9095599; doi:10.1038/s41467-022-30098-w)

Supplementary Information

Meta-analysis of sub-Saharan African studies provides insights into genetic architecture of lipid traits

Ananyo Choudhury[1], Jean-Tristan Brandenburg[1], Tinashe Chikowore[1,2], Dhriti Sengupta[1], Palwende Romuald Boua[1,3], Nigel J Crowther[4], Godfred Agongo[5,6], Gershim Asiki[7], F. Xavier Gómez-Olivé[8], Isaac Kisiangani[7], Eric Maimela[9], Matshane Masemola-Maphutha[10], Lisa K Micklesfield[2], Engelbert A Nonterah[5], Shane A Norris[2], Hermann Sorgho[3], Halidou Tinto[3], Stephen Tollman[8], Sarah E Graham[11], Cristen J Willer[11,12,13], AWI-Gen study* and H3Africa Consortium*, Scott Hazelhurst[1,14] and Michèle Ramsay[1,15]

1. Sydney Brenner Institute for Molecular Bioscience, Faculty of Health Sciences, University of the Witwatersrand, Johannesburg, South Africa
2. South African Medical Research Council/University of the Witwatersrand Developmental Pathways for Health Research Unit, Department of Paediatrics, School of Clinical Medicine, Faculty of Health Sciences, University of the Witwatersrand, Johannesburg
3. Clinical Research Unit of Nanoro, Institut de Recherche en Sciences de la Santè, Nanoro, Burkina Faso
4. Department of Chemical Pathology, National Health Laboratory Service, Faculty of Health Sciences, University of the Witwatersrand, Johannesburg, South Africa
5. Navrongo Health Research Centre, Ghana Health Service, Navrongo, Ghana
6. C.K. Tedam University of Technology and Applied Sciences, Navrongo, Ghana
7. African Population and Health Research Center, Nairobi, Kenya
8. MRC/Wits Rural Public Health and Health Transitions Research Unit (Agincourt), School of Public Health, Faculty of Health Sciences, University of the Witwatersrand, Johannesburg, South Africa
9. Department of Public Health, School of Health Care Sciences, Faculty of Health Sciences, University of Limpopo, Polokwane, South Africa
10. Department of Pathology and Medical Sciences, School of Health Care Sciences, Faculty of Health Sciences, University of Limpopo, Polokwane, South Africa
11. Department of Internal Medicine, Division of Cardiology, University of Michigan, Ann Arbor, MI 48109, USA
12. Department of Computational Medicine and Bioinformatics, University of Michigan, Ann Arbor, MI 48109, USA
13. Department of Human Genetics, University of Michigan, Ann Arbor, MI 48019, USA
14. School of Electrical and Information Engineering, University of the Witwatersrand, Johannesburg, South Africa
15. Division of Human Genetics, National Health Laboratory Service and School of Pathology, Faculty of Health Sciences, University of the Witwatersrand, Johannesburg, South Africa

Supplementary Figures

Supplementary Figure 1. **Distribution of the four lipid traits in participants from the six study-sites. a** LDL-C, **b** HDL-C, **c** Total-cholesterol and **d** Triglycerides. Violin plots for Males (M) (N=4,784) and females (F) (N=5,789) are shown in red and blue, respectively. The boxplot shows the median of the distribution (central line), with the top and bottom of the box indicating the third quartile (Q3) and the first quartile (Q1), respectively. Whiskers extend to minimum (Q1- 1.5 IQR) and maximum (Q1+ 1.5 IQR). The shape of the plot represent the probability distribution function.

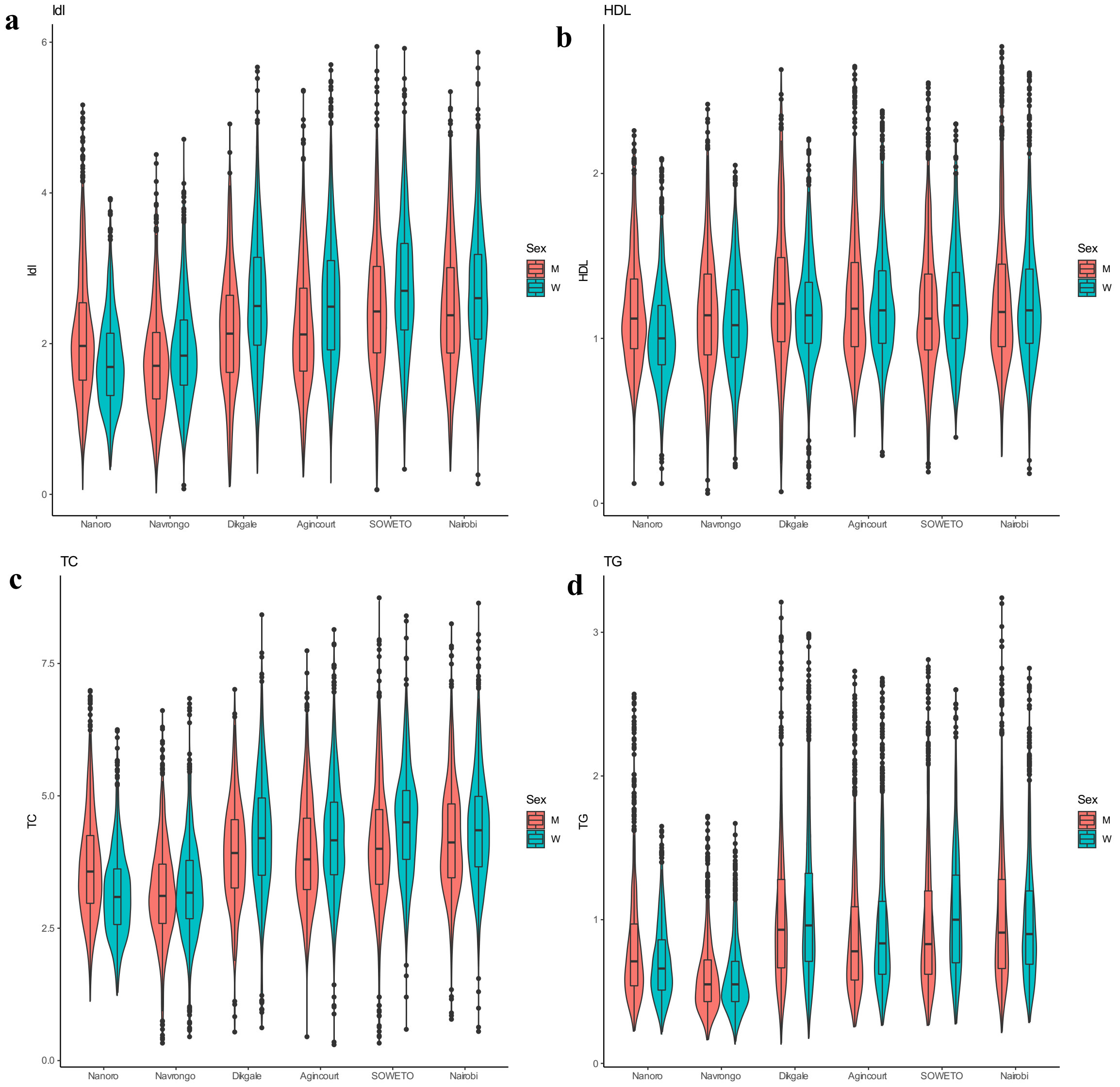

Supplementary Figure 2. Population structure in the AWI-Gen cohort. a Principal Components (PC) analysis plot showing population structure based partitioning of the AWI-Gen samples (*N*=10,603). Participants are coloured according to the collection site. Navrongo and Nanoro are located in West Africa, Agincourt, Dikgale and Soweto in South Africa and Nairobi in East Africa. **b** A composite plot showing the first 20 PCs for representative ethnolinguistic groups included among our samples. Each group is shown in a different colour. Based on this and similar plots, we selected the first 8 PCs to be most relevant for analysis of the full dataset and up to first 4 PCs to be relevant for East, West and South African subsets and were used as covariates in the respective GWASs.

a

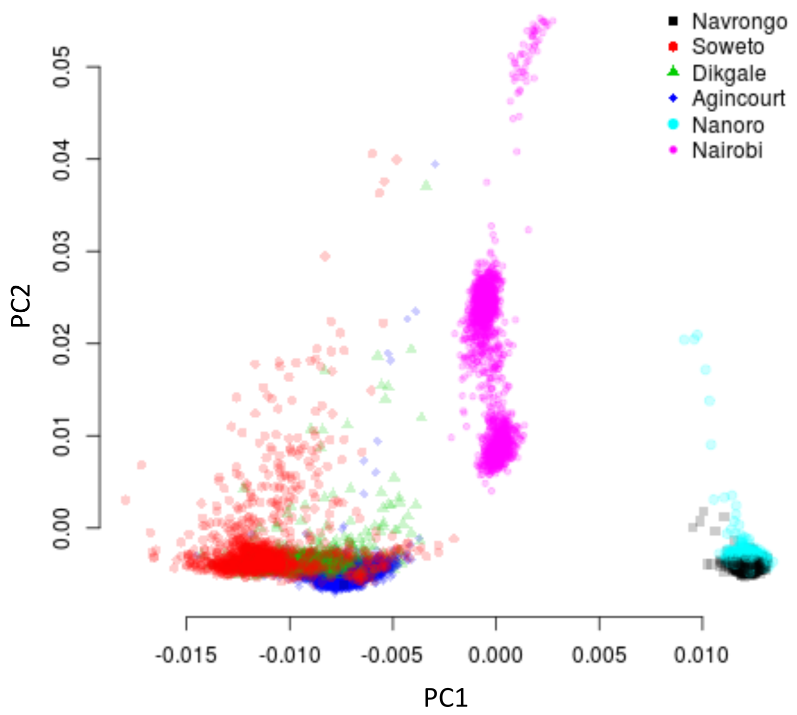

b

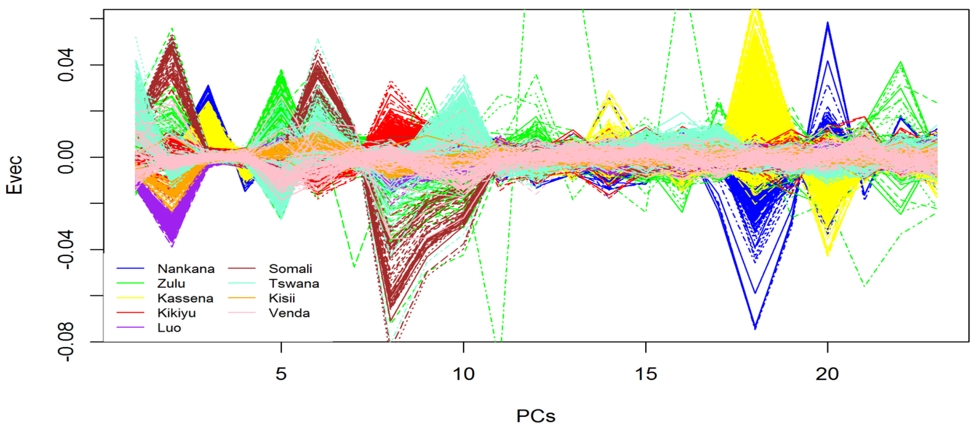

Supplementary Figure 3. A composite QQ plot for associations detected in the Stage 1 GWAS for total cholesterol (cholesterol), LDL-C (ldl), HDL-C (hdl) and triglycerides. Respective lambda (λ) values are shown within brackets.

.

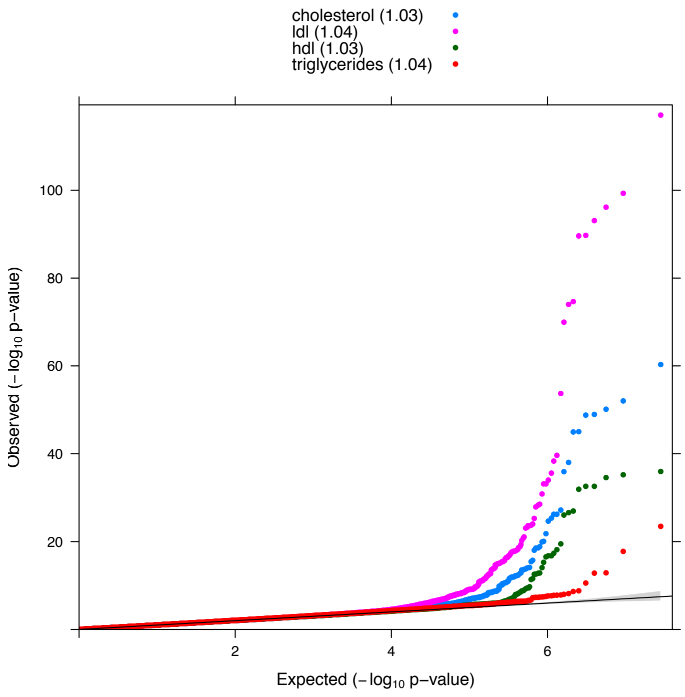

Supplementary Figure 4. Genome-wide associations for total cholesterol. Miami plot showing summary data for Stage 1 GWAS (AWI-Gen, downward facing, *N*=10,603) and Stage 2 GWAS (meta-analysis of AWI-Gen and four African cohorts, upward facing, *N*=23,718). *P*-values (two-tailed, not adjusted for multiple comparisons, calculated using BOLT-LMM for Stage 1 GWAS and METASOFT for Stage 2 GWAS) are truncated at 10^{-20} for clarity. The red horizontal lines show the genome-wide significance threshold ($5x10^{-8}$) and SNPs with *P*-values below this threshold are shown in orange.

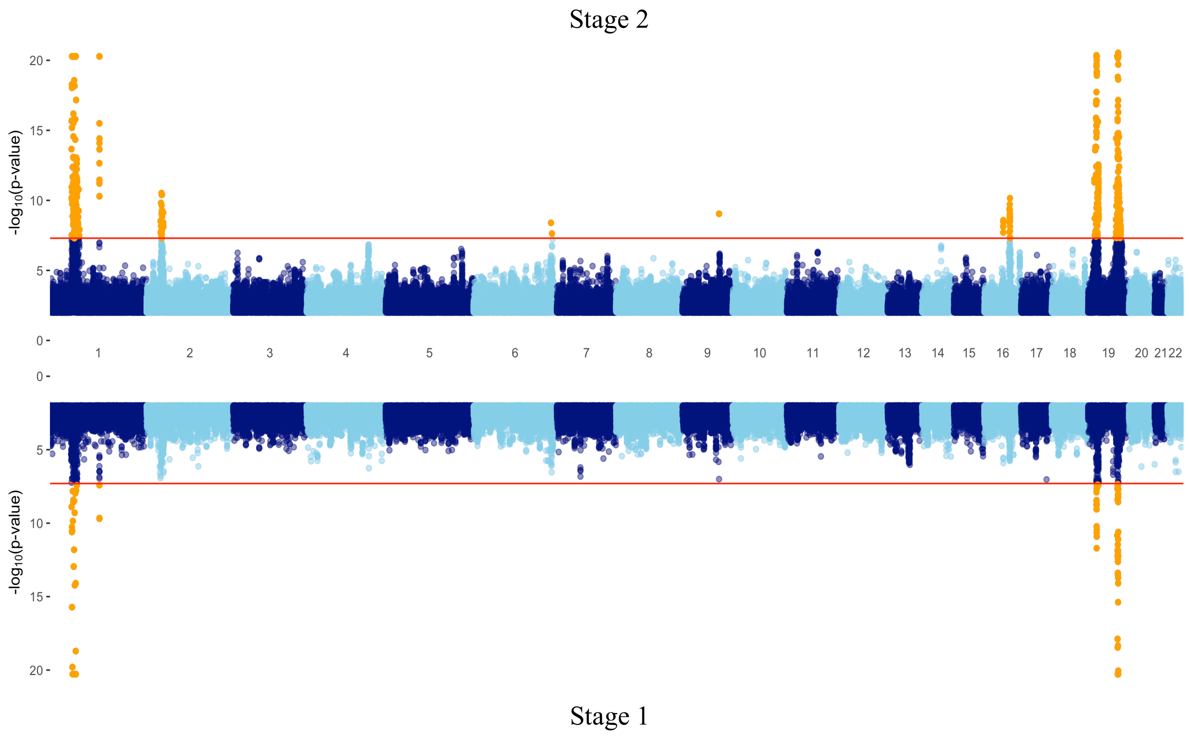

Supplementary Figure 5. Genome-wide associations for HDL-C. Miami plot showing summary data for Stage 1 GWAS (AWI-Gen, downward facing, *N*=10,603) and Stage 2 GWAS (meta-analysis of AWI-Gen and four African cohorts, upward facing, *N*=23,718). *P*-values (two-tailed, not adjusted for multiple comparisons, calculated using BOLT-LMM for Stage 1 GWAS and METASOFT for Stage 2 GWAS) are truncated at 10^{-20} for clarity. The red horizontal lines show the genome-wide significance threshold ($5x10^{-8}$) and SNPs with *P*-values below this threshold are shown in orange.

Stage 2

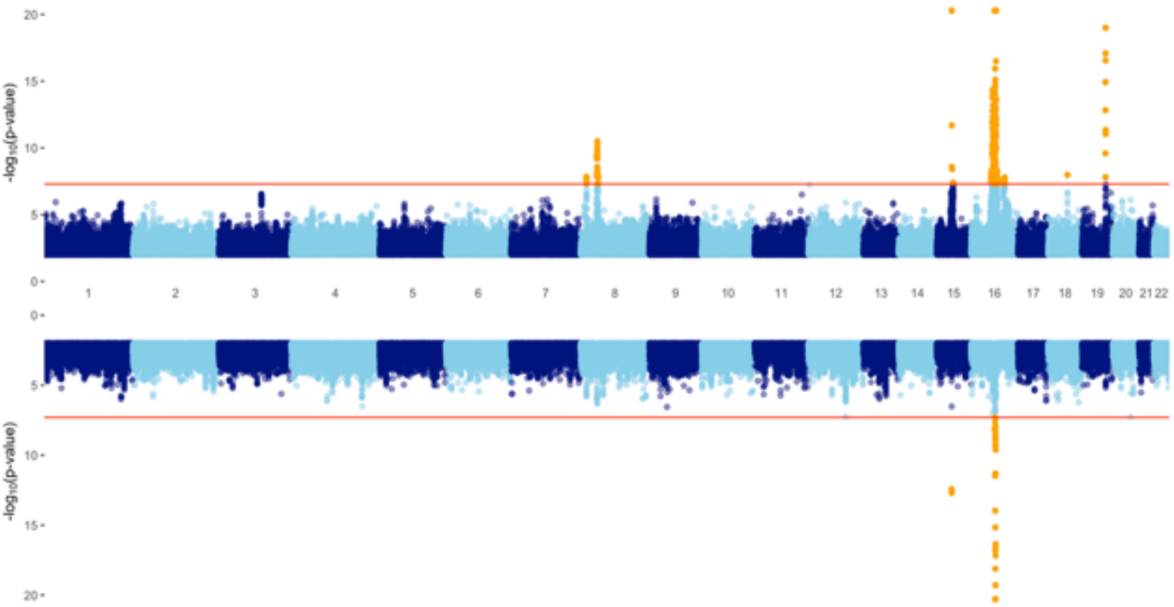

Stage 1

Supplementary Figure 6. Genome-wide associations for Triglycerides. Miami plot showing summary data for Stage 1 GWAS (AWI-Gen, downward facing, *N*=10,603) and Stage 2 GWAS (meta-analysis of AWI-Gen and four African cohorts, upward facing, *N*=23,718). *P*-values (two-tailed, not adjusted for multiple comparisons, calculated using BOLT-LMM for Stage 1 GWAS and METASOFT for Stage 2 GWAS) are truncated at 10^{-20} for clarity. The red horizontal lines show the genome-wide significance threshold ($5x10^{-8}$) and SNPs with *P*-values below this threshold are shown in orange. The novel signal near the *FHIT* gene is highlighted by a dotted rectangle.

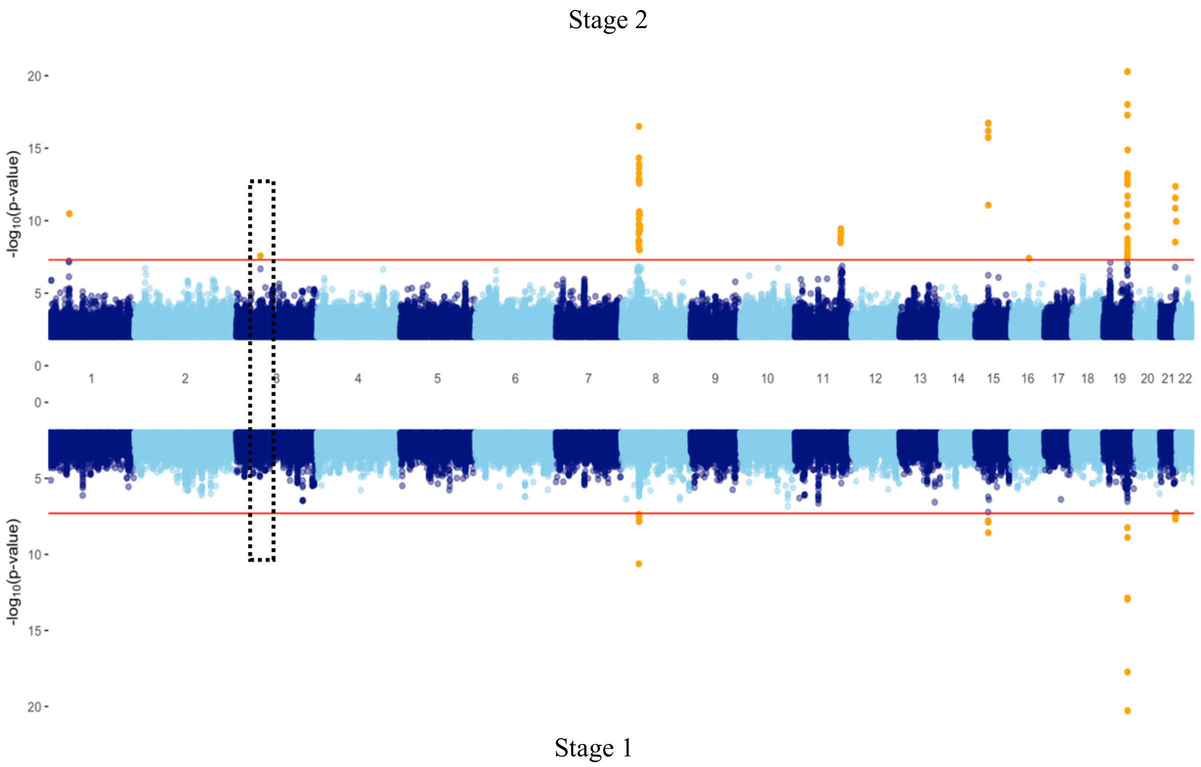

Supplementary Figure 7. Locuszoom plots showing distribution of association signals for LDL-C around the *PCSK9* region. a Stage 1 GWAS. **b** Stage 2 GWAS.

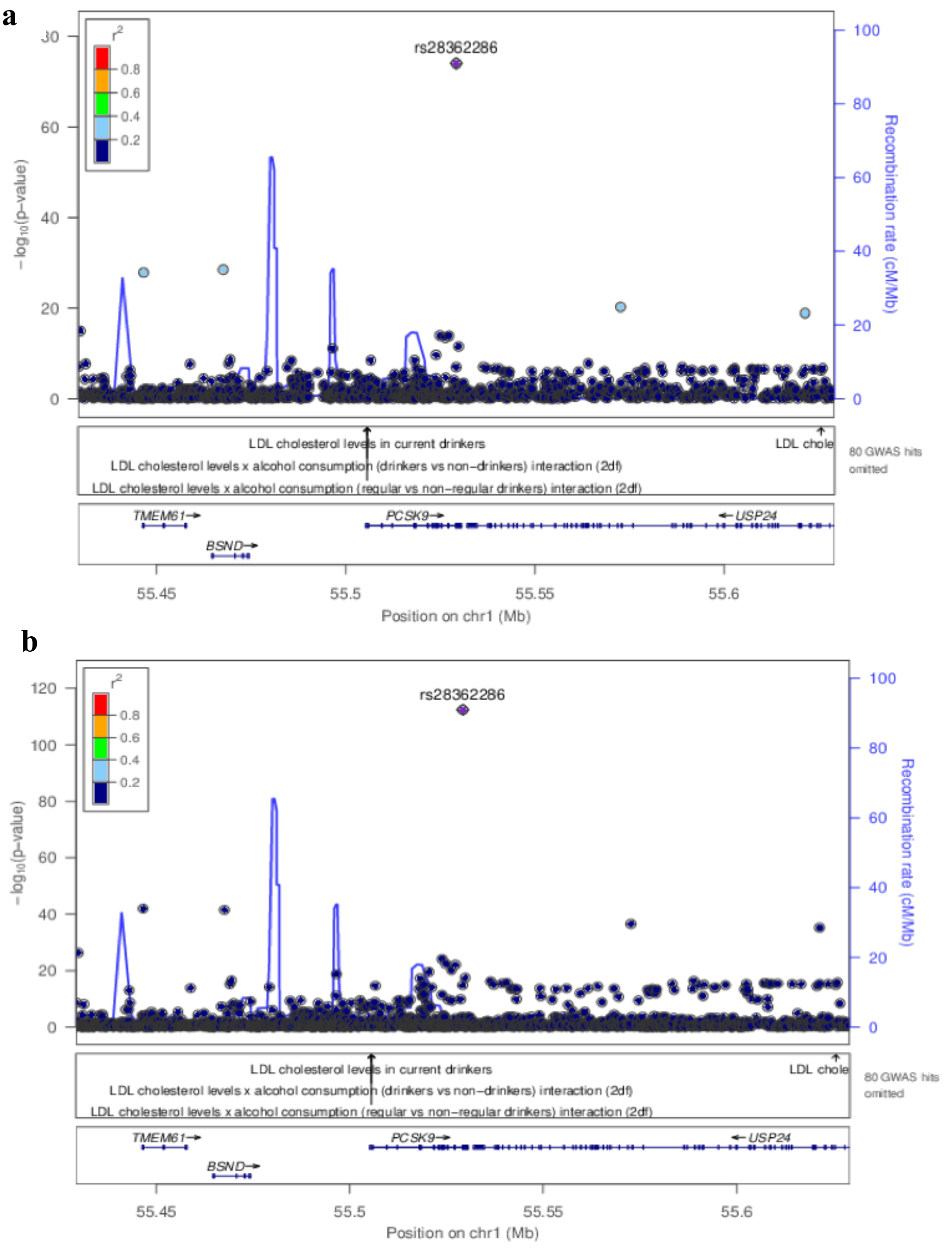

Supplementary Figure 8. Locuszoom plots showing associations for LDL cholesterol in the AWI-Gen GWAS (a-d) and the corresponding regions in the Prins et al. 2017 study (e-h). Locuszoom plot for the other three LDL-cholesterol associations in the AWI-Gen study are presented in **Fig. 3** and **Supplementary Fig. 7**.

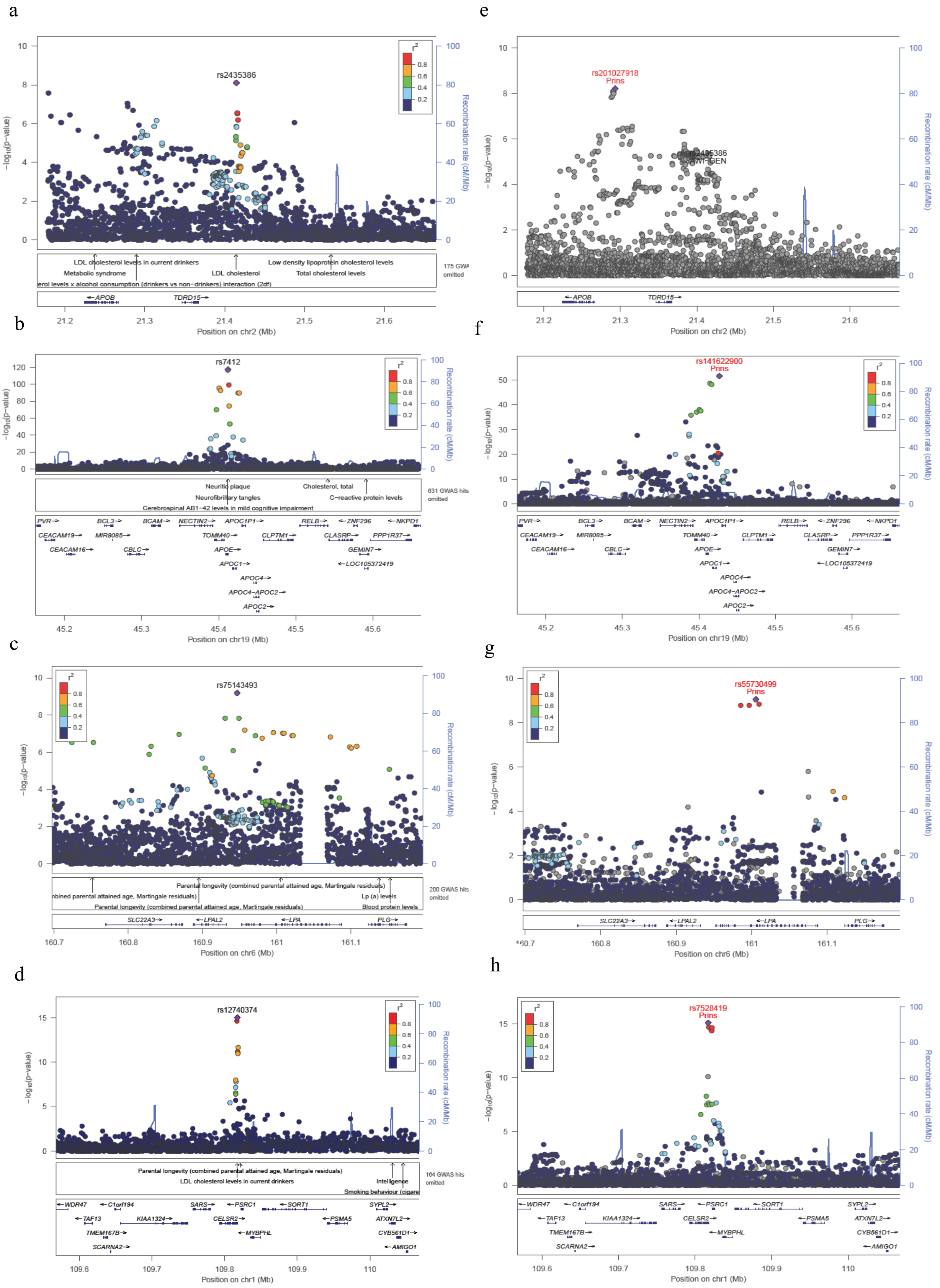

Supplementary Figure 9. Locuszoom plots showing associations for HDL cholesterol in the AWI-Gen GWAS (a, b) and the corresponding regions in the Prins et al. 2017 study (c, d).

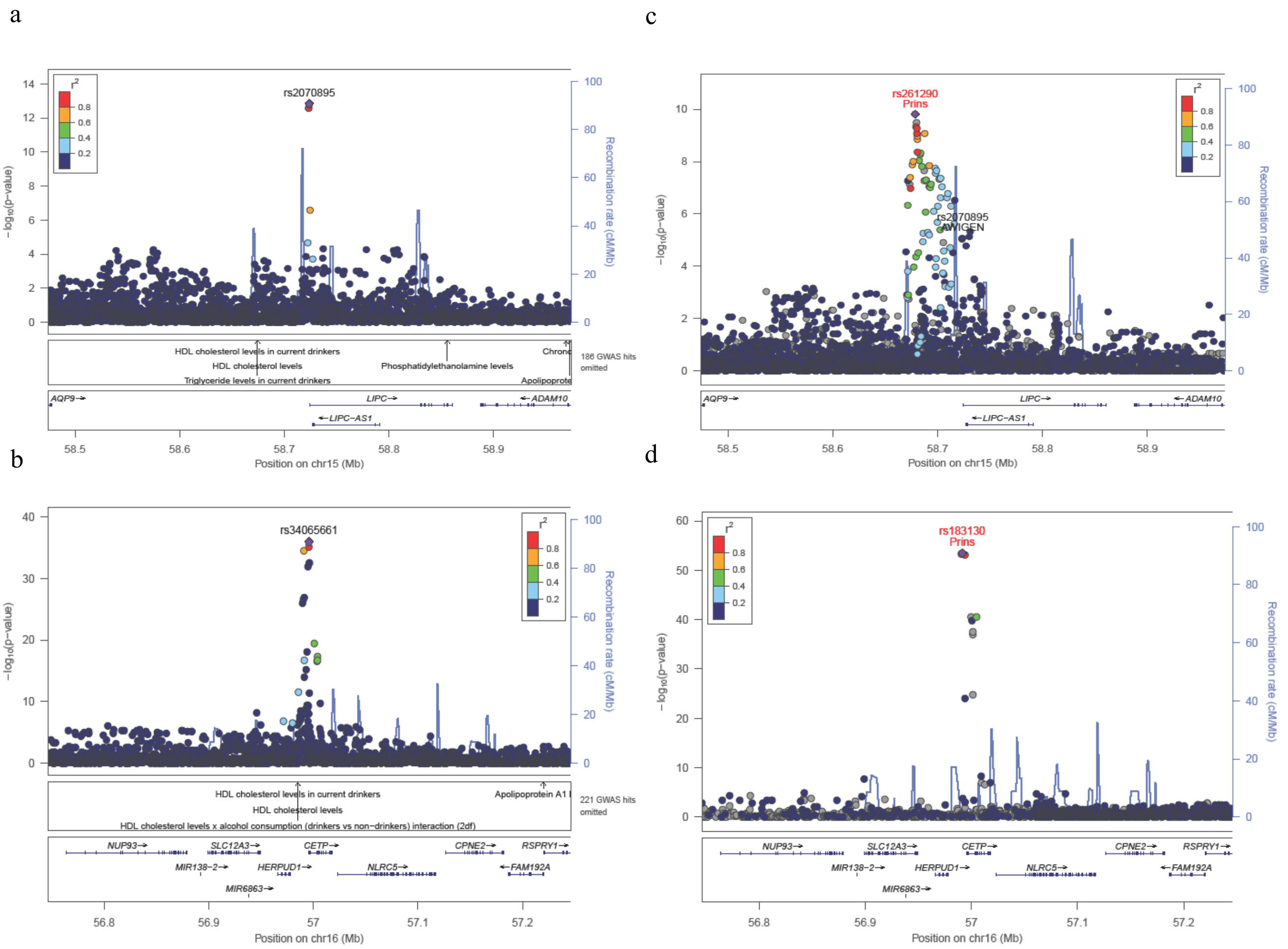

Supplementary Figure 10. Locuszoom plots showing associations for Triglycerides in the AWI-Gen GWAS (a-d) and the corresponding regions in the Prins et al. 2017 study (e-h).

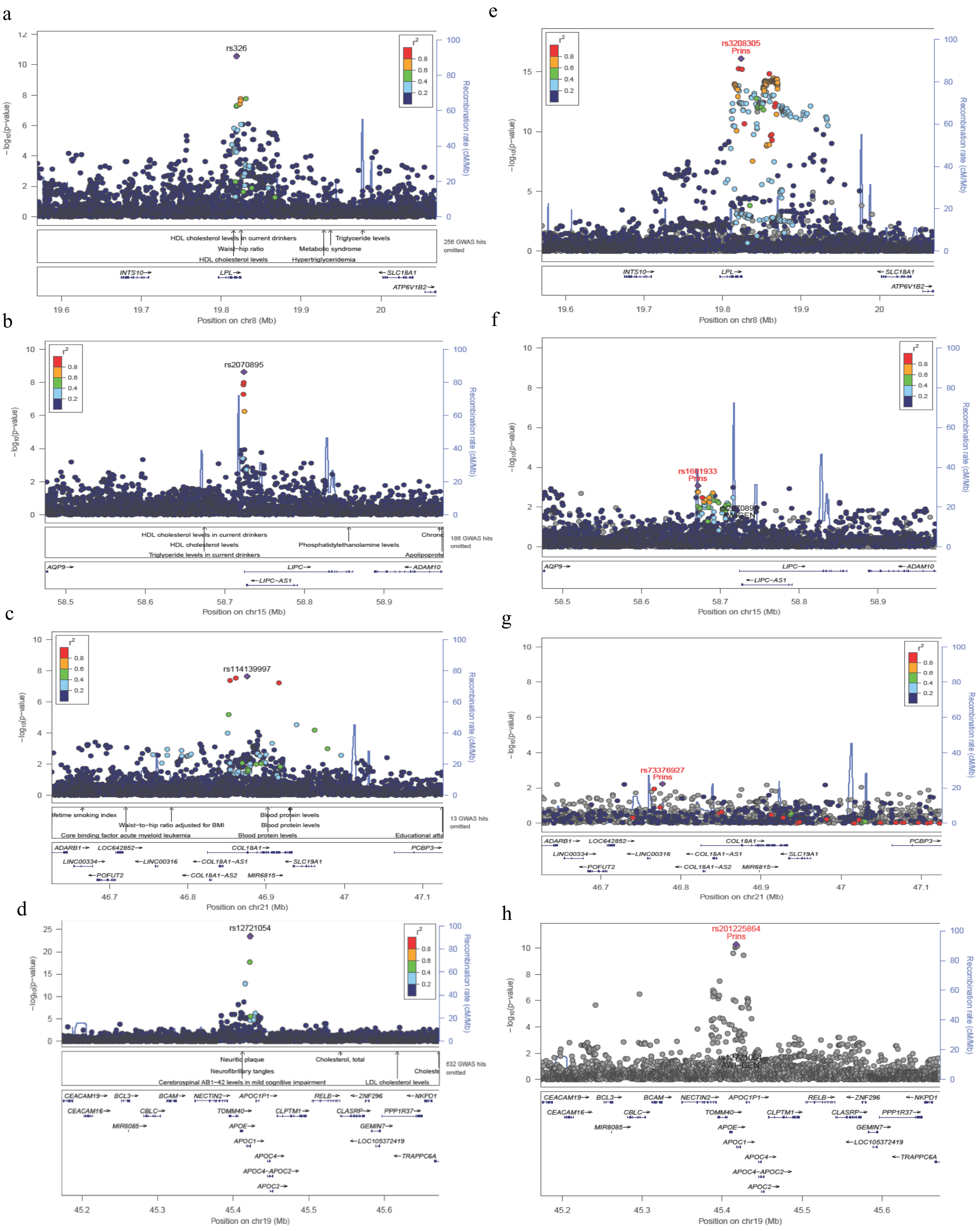

Supplementary Figure 11. Locuszoom plots showing associations for Total cholesterol in the AWI-Gen GWAS (a-d) and the corresponding regions in the Prins et al. 2017 study (e-h).

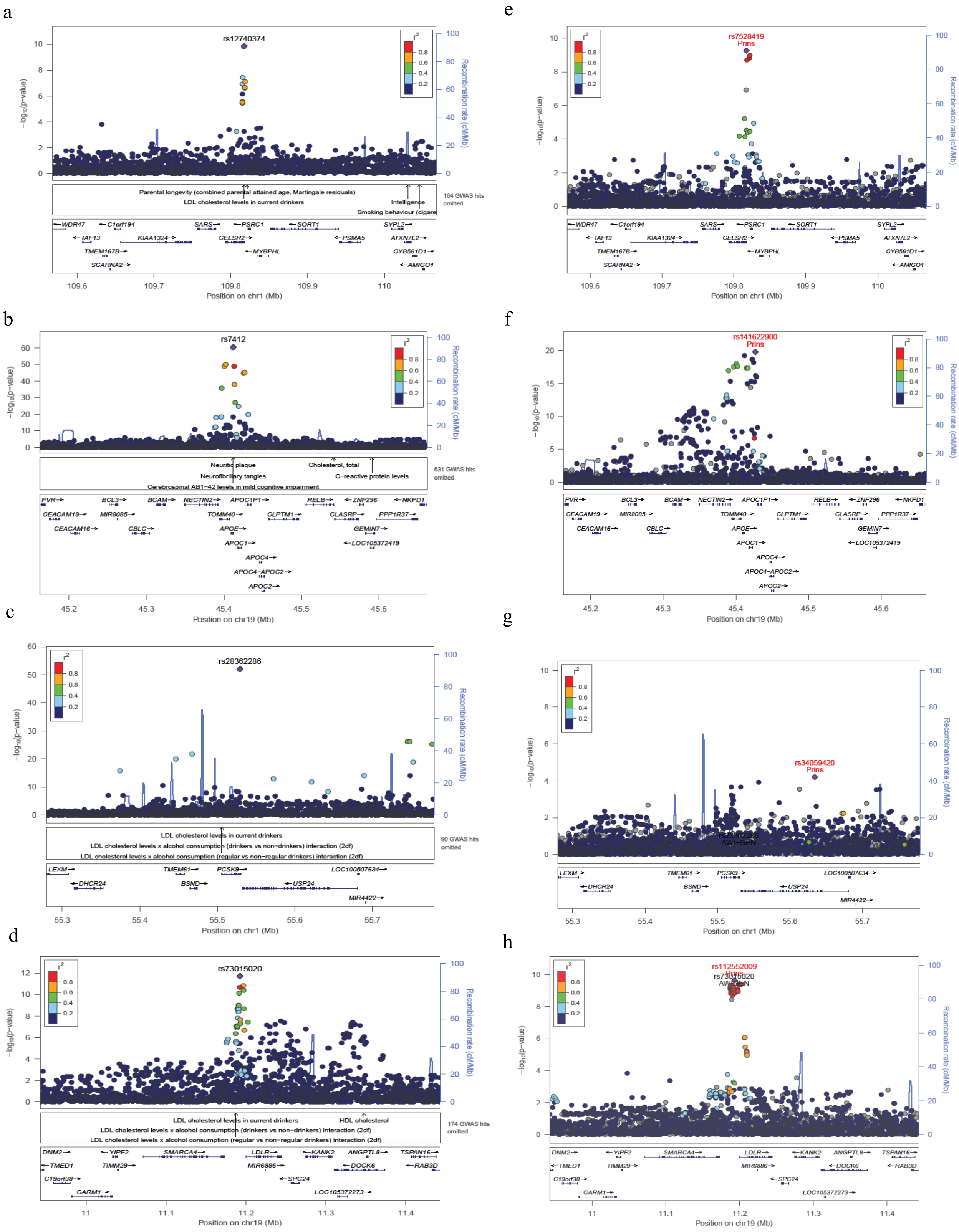

Supplementary Figure 12. A composite QQ plot for the associations detected in the Stage 2 GWAS (meta-analysis of AWI-Gen and four other African cohorts) for total cholesterol (cholesterol), LDL-C (ldl), HDL-C (hdl) and triglycerides. Respective lambda (λ) values are shown within brackets.

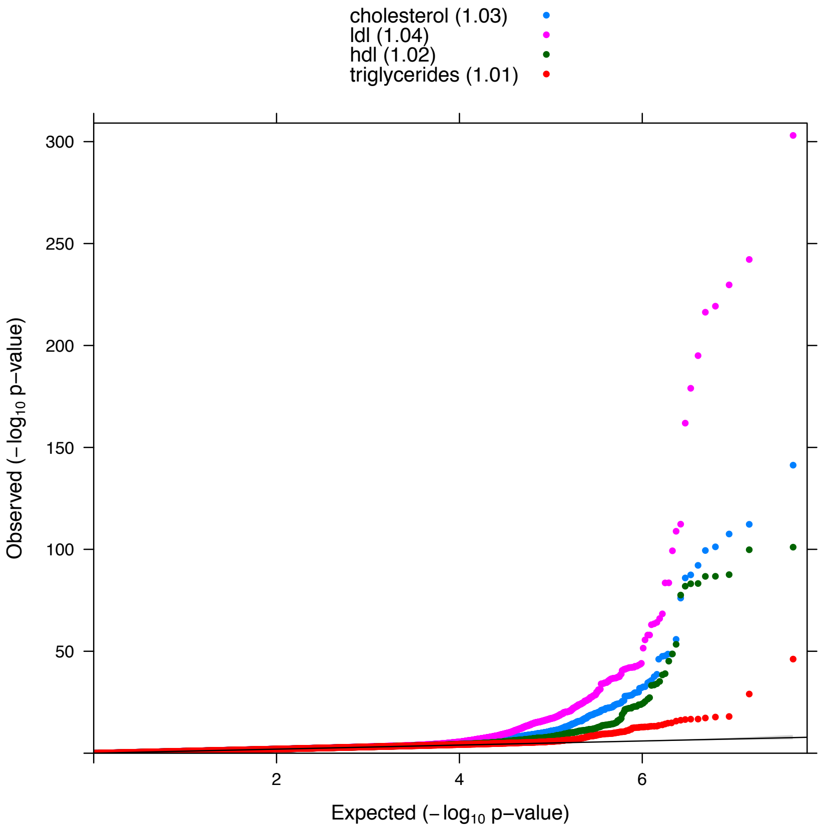

Supplementary Figure 13. Locuszoom plot summarising associations for LDL-C around the *LPA* region. a Stage 1 GWAS. **b** Stage 2 GWAS.

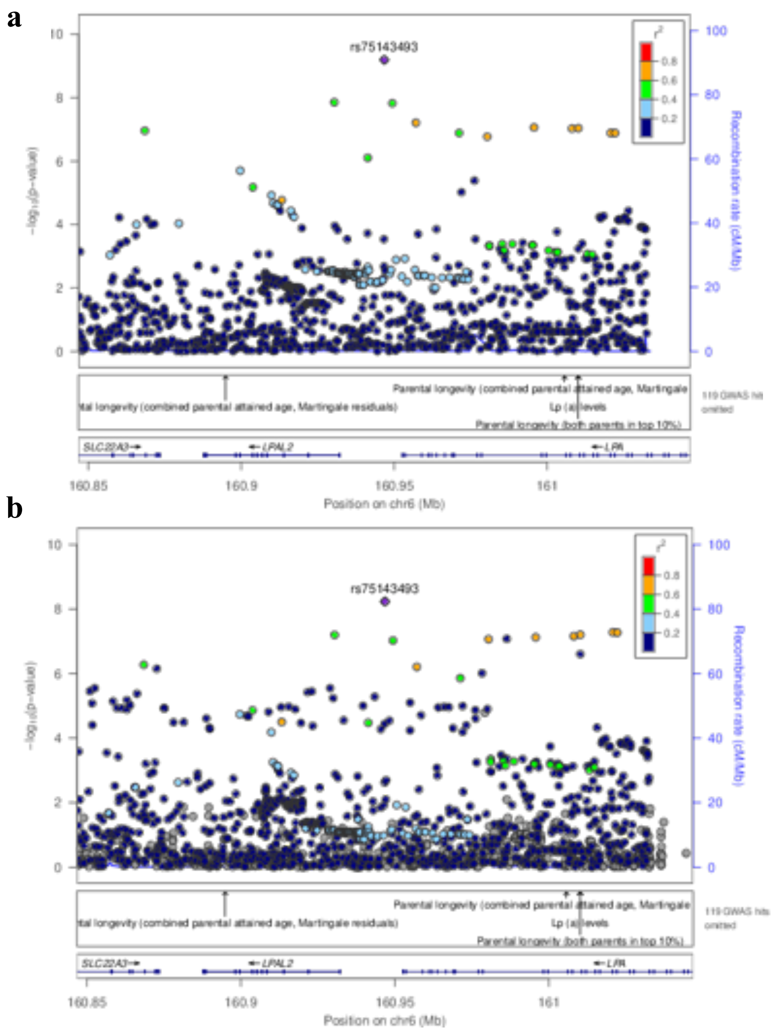

Supplementary Figure 14. Transferability of previous lipid associations to various African GWASs. a HDL-C associations reported in the GLGC study (Willer et al. 2013). **b** HDL-C associations reported in the PAGE study (Wojcik et al. 2019). **c** Total cholesterol (TC) associations reported in the GLGC study. **d** TC associations reported in the PAGE study. **e** Triglyceride (TG) associations reported in the GLGC study. **f** TG associations reported in the PAGE study. Proportion of signals replicated at the genome wide significance threshold are shown in deep blue (F-GW), at *P*-value<$5X10^{-4}$ are shown in dark green (F_RepThr) and at the nominal threshold (*P*-value<0.05) are shown in orange (F_NT). The signals from GLGC study were partitioned based on *P*-value into Very Strong (*P*-value<10^{-100}) indicated by the suffix VS and a green background, Strong (10^{-20}<*P*-value >10^{-100}) indicated by the suffix S and blue background, and moderate ($5X10^{-8}$<*P*-value >10^{-20}) indicated by the suffix M and a grey background. The replication of signals in each category are shown separately. For PAGE only two categories, Strong (S) (*P*-value<10^{-20}) and moderate (M) were considered ($5X10^{-8}$<*P*-value >10^{-20}). The African replication datasets used in the analysis are - Stage 2 GWAS (Meta, *N*=23,718), Stage 1 GWAS (AWI, *N*=10,603), Uganda Genome Resource (UG, *N*=6,407) study, Africa-America Diabetes Mellitus (AADM, *N*= 4,116) study, Durban Diabetes study (DDS, *N*=1,117) and Durban case control (DCC, *N*=1,755) study.

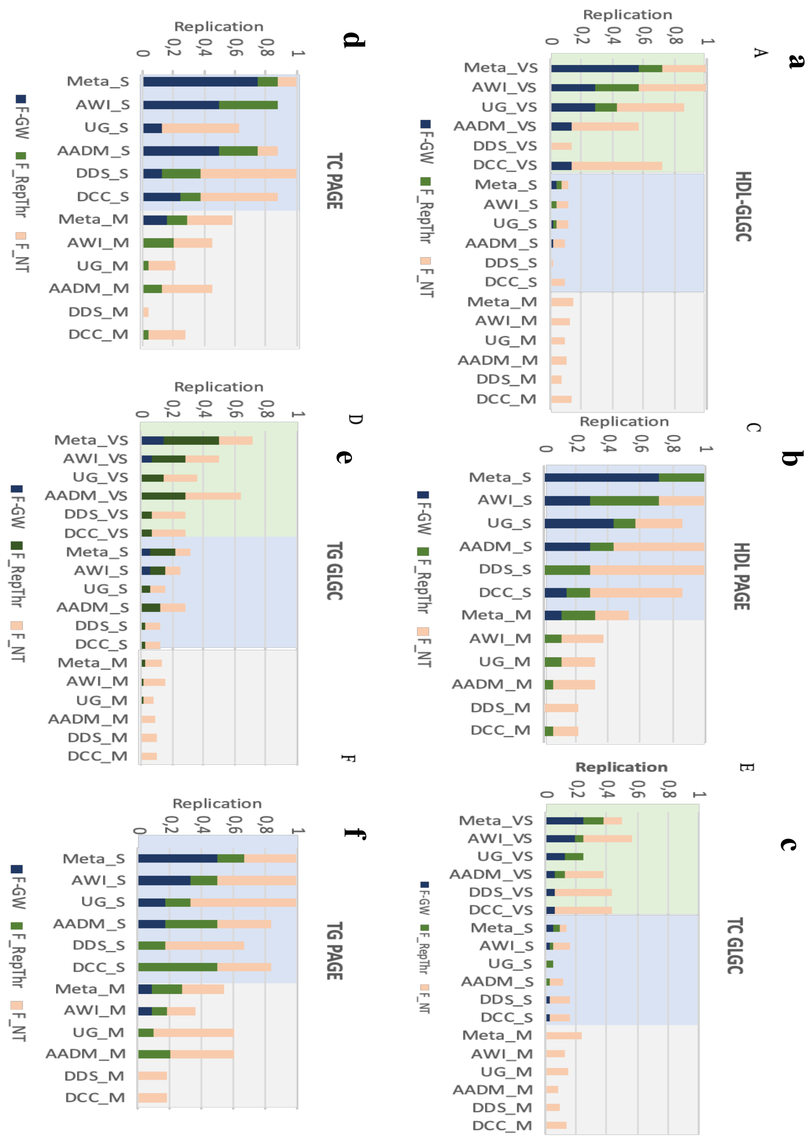

Supplementary Figure 15. Effect size heterogeneity in LDL-C signals. Forest plot showing Beta +/- 95% confidence interval of the lead SNPs detected in the LDL-C meta-analysis. The rsIDs are shown in grey box at the top of each plot. Estimates for the meta-analysis are shown in red (with values) and other GWASs are shown in black. These include Stage 1 GWAS (AWI-All, *N*=10,603), Stage 2 GWAS (Meta, *N*=23,718), Uganda Genome Resource (UGR, *N*= 6,407) study, Africa-America Diabetes Mellitus (AADM, *N*= 4,116) study, Durban Diabetes (DDS, *N*=1,117) study and Durban case control (DCC, *N*= 1,475) study. The three regional AWI-Gen datasets- AWI-South (AWI-Gen South African samples, *N*=5,085), AWI-West (AWI-Gen West African samples, *N*=3,763) and AWI-East (AWI-Gen East African samples, *N*=1,755) are shown for comparative purposes.

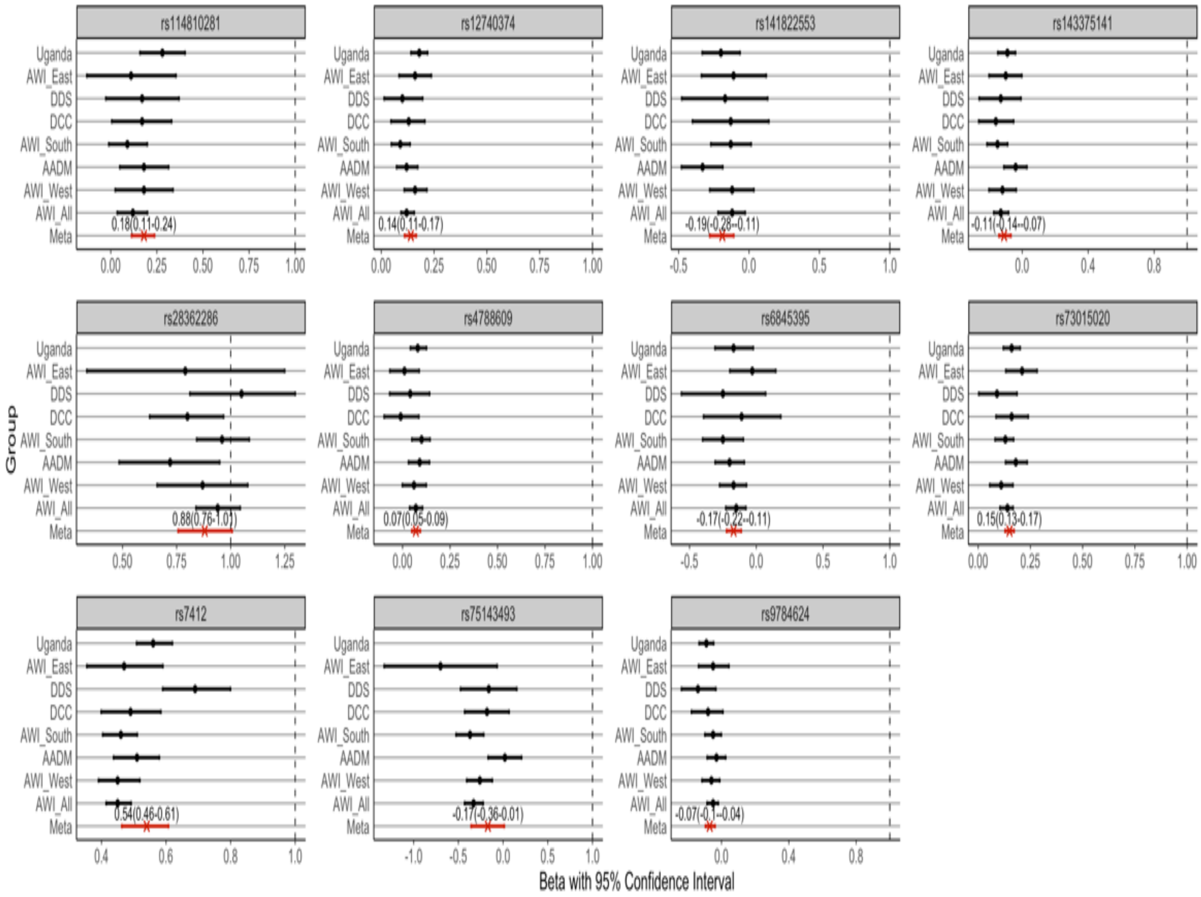

Supplementary Figure 16. Effect size heterogeneity in total cholesterol signals. Forest plot showing Beta +/- 95% confidence interval of each the lead SNPs in the total cholesterol meta-analysis. The rsIDs are show in the grey box at the top of each plot. Estimates for the meta-analysis are shown in red (with values) and other GWASs are shown in black. These include Stage 1 GWAS (AWI-All, *N*=10,603), Stage 2 GWAS (Meta, *N*=23,718), Uganda Genome Resource (UGR, *N*= 6,407) study, Africa-America Diabetes Mellitus (AADM, *N*= 4,116) study, Durban Diabetes (DDS, *N*=1,117) study and Durban case control (DCC, *N*= 1,475) study. The three regional AWI-Gen datasets- AWI-South (AWI-Gen South African samples, *N*=5,085), AWI-West (AWI-Gen West African samples, *N*=3,763) and AWI-East (AWI-Gen East African samples, *N*=1,755) are shown for comparative purposes.

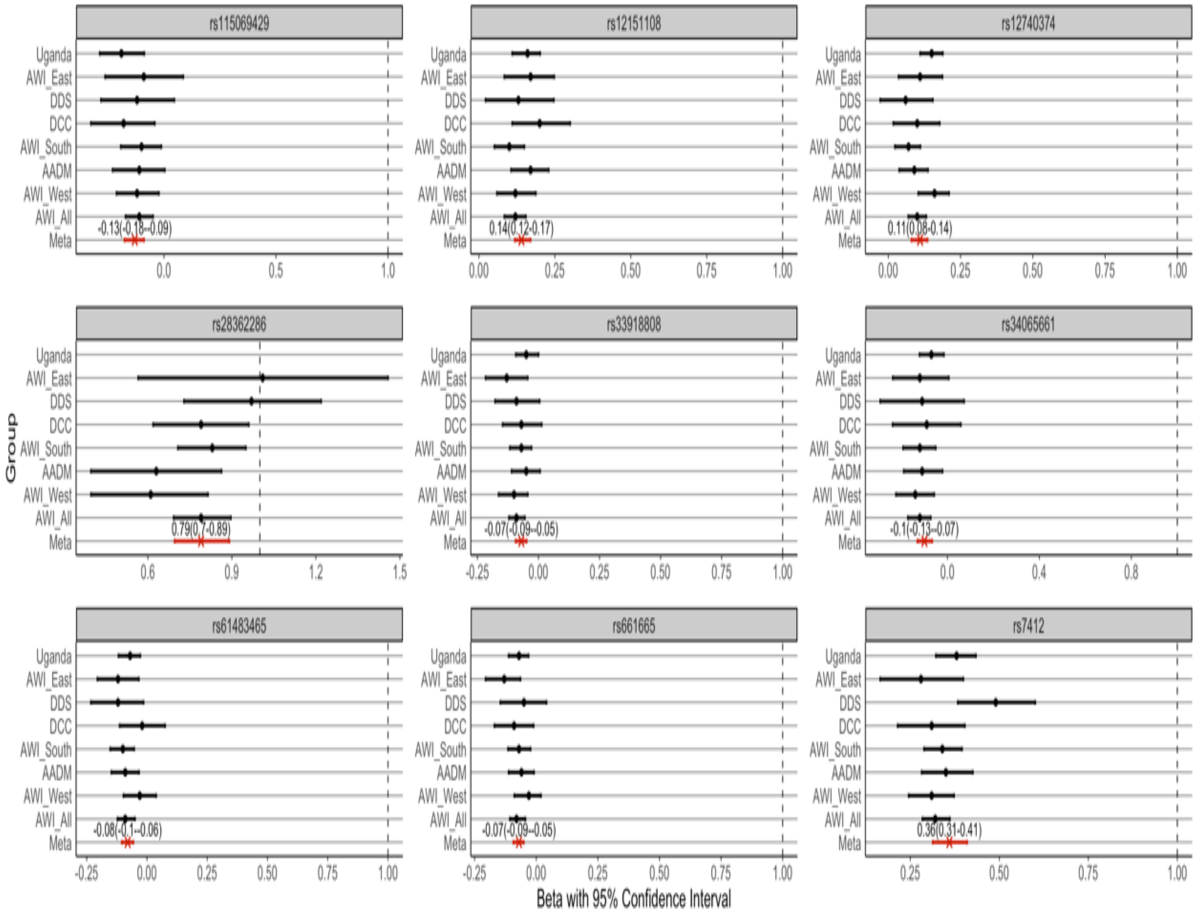

Supplementary Figure 17. Effect size heterogeneity in HDL-C signals. Forest plot showing the Beta +/- 95% confidence interval of the lead SNPs detected in the HDL-cholesterol meta-analysis. The rsIDs are shown in grey box at the top of each plot. Estimates for the meta-analysis are shown in red (with values) and other GWASs are shown in black. These include Stage 1 GWAS (AWI-All, *N*=10,603), Stage 2 GWAS (Meta, *N*=23,718), Uganda Genome Resource (UGR, *N*= 6,407) study, Africa-America Diabetes Mellitus (AADM, *N*= 4,116) study, Durban Diabetes (DDS, *N*=1,117) study and Durban case control (DCC, *N*= 1,475) study. The three regional AWI-Gen datasets- AWI-South (AWI-Gen South African samples, *N*=5,085), AWI-West (AWI-Gen West African samples, *N*=3,763) and AWI-East (AWI-Gen East African samples, *N*=1,755) are shown for comparative purposes.

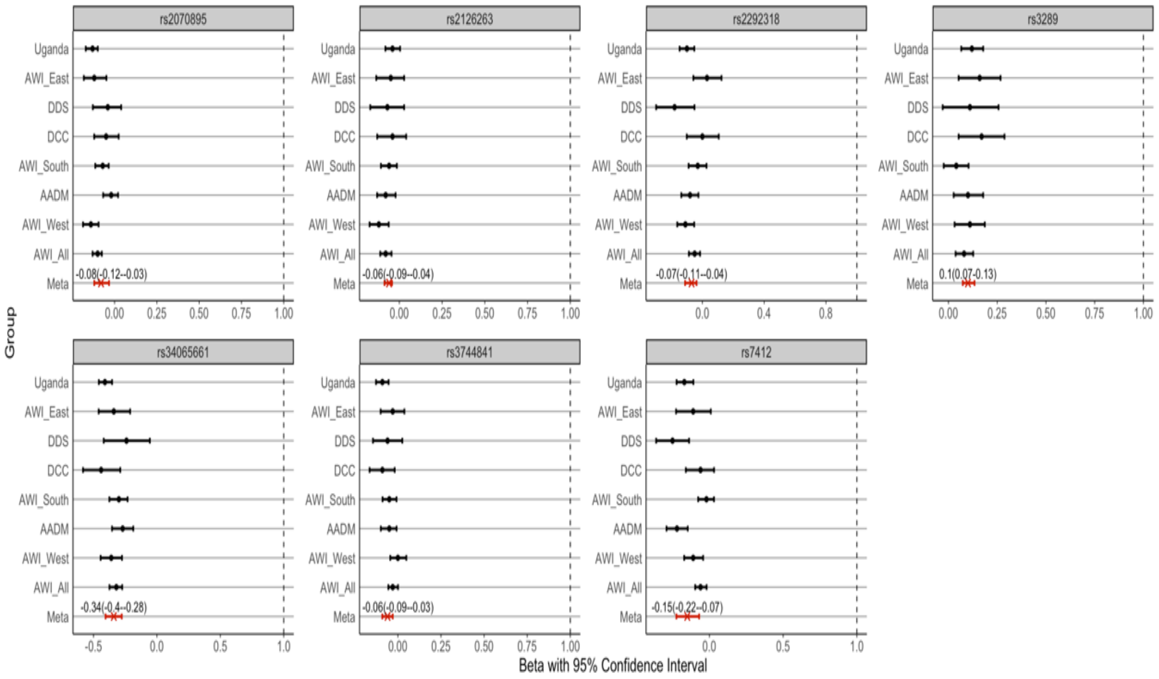

Supplementary Figure 18. Effect size heterogeneity in Triglyceride signals. Forest plot showing Beta +/- 95% confidence interval of lead SNPs detected in the HDL-cholesterol meta-analysis. The rsIDs are shown in grey box at the top of each plot. Estimates for the meta-analysis are shown in red (with values) and other GWASs are shown in black. These include Stage 1 GWAS (AWI-All, *N*=10,603), Stage 2 GWAS (Meta, *N*=23,718), Uganda Genome Resource (UGR, *N*= 6,407) study, Africa-America Diabetes Mellitus (AADM, *N*= 4116) study, Durban Diabetes (DDS, *N*=1,117) study and Durban case control (DCC, *N*= 1,475) study. The three regional AWI-Gen datasets- AWI-South (AWI-Gen South African samples, *N*=5,085), AWI-West (AWI-Gen West African samples, *N*=3,763) and AWI-East (AWI-Gen East African samples, *N*=1,755) are shown for comparative purposes.

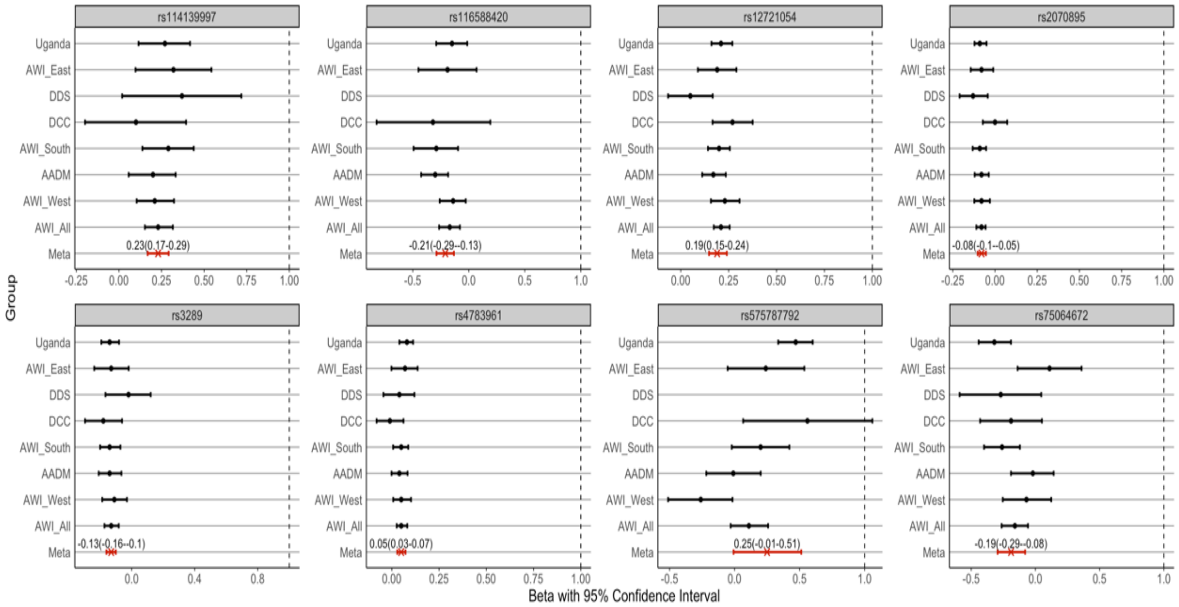

Supplement: Supplementary file 1 — Supplementary Information [file 41467_2022_30098_MOESM1_ESM.pdf]
