# Supplementary material for: Meta-analysis of sub-Saharan African studies provides insights into genetic architecture of lipid traits
Source: Nat Commun. 2022 May 11;13:2578. doi: 10.1038/s41467-022-30098-w (PMC9095599; doi:10.1038/s41467-022-30098-w)
Supplement: Supplementary file 3 — Description of Additional Supplementary Files [file 41467_2022_30098_MOESM3_ESM.pdf]

Description of Supplementary Datasets

File Name: Supplementary Data 1
Description: Characteristics of the individual AWI-Gen study sites and the full cohort.

File Name: Supplementary Data 2
Description: Comparison of associations detected using joint analysis (of the South, East and West African data) and meta-analysis (of the summary statistics from the three region specific GWASs).

File Name: Supplementary Data 3
Description: Extended information for associations detected in the Stage 1 GWAS.

File Name: Supplementary Data 4
Description: Suggestive associations (P-value<$1X10^{-6}$) detected in Stage 1 GWAS for the four lipid traits.

File Name: Supplementary Data 5
Description: Genome wide significant associations detected in geographic region-specific GWAS.

File Name: Supplementary Data 6
Description: Extended information for associations detected in the Stage 2 GWAS.

File Name: Supplementary Data 7
Description: List of enriched gene sets (after Bonferroni Correction) in the Stage 1 and Stage 2 GWAS association results for the four lipid traits.

File Name: Supplementary Data 8
Description: Predictivity of PRS models in the AWI-Gen cohort.

File Name: Supplementary Data 9
Description: Minor allele frequency of association signals detected in the Stage 2 GWAS, in the constituent African cohorts

File Name: Supplementary Data 10
Description: Look-up for three novel association signals detected in AWI-Gen study in the GLGC African American and transethnic datasets.
