# Supplementary material for: Meta-analysis of sub-Saharan African studies provides insights into genetic architecture of lipid traits
Source: Nat Commun. 2022 May 11;13:2578. doi: 10.1038/s41467-022-30098-w (PMC9095599; doi:10.1038/s41467-022-30098-w)
Supplement: Supplementary file 5 — Reporting Summary [file 41467_2022_30098_MOESM5_ESM.pdf]

nature research

Corresponding author(s): Michele Ramsay; Ananyo Choudhury

Last updated by author(s): 22/03/22

Reporting Summary

Nature Research wishes to improve the reproducibility of the work that we publish. This form provides structure for consistency and transparency in reporting. For further information on Nature Research policies, see our Editorial Policies and the Editorial Policy Checklist.

Statistics

For all statistical analyses, confirm that the following items are present in the figure legend, table legend, main text, or Methods section.

n/a	Confirmed	
☐	☒	The exact sample size (*n*) for each experimental group/condition, given as a discrete number and unit of measurement
☒	☐	A statement on whether measurements were taken from distinct samples or whether the same sample was measured repeatedly
☐	☒	The statistical test(s) used AND whether they are one- or two-sided<br>*Only common tests should be described solely by name; describe more complex techniques in the Methods section.*
☐	☒	A description of all covariates tested
☐	☒	A description of any assumptions or corrections, such as tests of normality and adjustment for multiple comparisons
☐	☒	A full description of the statistical parameters including central tendency (e.g. means) or other basic estimates (e.g. regression coefficient) AND variation (e.g. standard deviation) or associated estimates of uncertainty (e.g. confidence intervals)
☐	☒	For null hypothesis testing, the test statistic (e.g. *F*, *t*, *r*) with confidence intervals, effect sizes, degrees of freedom and *P* value noted<br>*Give P values as exact values whenever suitable.*
☒	☐	For Bayesian analysis, information on the choice of priors and Markov chain Monte Carlo settings
☒	☐	For hierarchical and complex designs, identification of the appropriate level for tests and full reporting of outcomes
☐	☒	Estimates of effect sizes (e.g. Cohen's *d*, Pearson's *r*), indicating how they were calculated

Our web collection on statistics for biologists contains articles on many of the points above.

Software and code

Policy information about availability of computer code

Data collection	No software was used for data collection.
Data analysis	PLINK 1.9, BOLT-LMMs, EIGENSTRAT, LDSC software, METASOFT v 2.01, FINEMAP, CAVIAR, PAINTOR; H3A GWAS pipeline (https://github.com/h3abionet/h3agwas) , GCTA-COJO; FUMA (fuma.ctglab.nl/fuma.ctglab.nl/), LocusZoom, Causal DB (http://mulinlab.org/causaldb/) and PhenoScanner (http://www.phenoscanner.medschl.cam.ac.uk/).

For manuscripts utilizing custom algorithms or software that are central to the research but not yet described in published literature, software must be made available to editors and reviewers. We strongly encourage code deposition in a community repository (e.g. GitHub). See the Nature Research guidelines for submitting code & software for further information.

Data

Policy information about availability of data

All manuscripts must include a data availability statement. This statement should provide the following information, where applicable:

- Accession codes, unique identifiers, or web links for publicly available datasets
- A list of figures that have associated raw data
- A description of any restrictions on data availability

The full dataset generated in this study is in the EGA [https://ega-archive.org/] database under the study accession code EGA00001002482 [https://ega-archive.org/studies/EGAS00001002482]. This includes the phenotype dataset EGAD00001006425 [https://ega-archive.org/datasets/EGAD00001006425] and the genotype dataset EGAD00010001996 [https://ega-archive.org/datasets/EGAD00010001996]. These datasets are available subject to controlled access through the Data and Biospecimen Access Committee of the H3Africa Consortium. The processed data generated in this study are provided in Supplementary Information and Supplementary Data. All data that support the findings of this study are available from the corresponding authors on request. Publicly available datasets included in the study are the following: 1000 Genomes Project Phase 3 (ftp://ftp.1000genomes.ebi.ac.uk/vol1/ftp), UGR meta-analysis summary statistics, GLGC summary

Statistics, PAGE consortium summary statistics available at GWAS Catalog (https://www.ebi.ac.uk/gwas/) and GNomAD (https://gnomad.broadinstitute.org/).

Field-specific reporting

Please select the one below that is the best fit for your research. If you are not sure, read the appropriate sections before making your selection.

- [x] Life sciences
- [ ] Behavioural & social sciences
- [ ] Ecological, evolutionary & environmental sciences

For a reference copy of the document with all sections, see nature.com/documents/nr-reporting-summary-flat.pdf

Life sciences study design

All studies must disclose on these points even when the disclosure is negative.

Sample size	The AWI-Gen study collected DNA and bio markers from about 12,000 participants across 6 centers in 4 African countries. About 11,000 of these participants were genotyped and included in the study. This was a population cross-sectional study and did not exclude or include participants on the basis of specific traits. No specific calculation was performed to determine sample sizes. A ballpark figure of 2000 samples per site was determined on the basis of infrastructure and resources available at the 6 centers. In terms of sample size this the largest GWAS for lipid traits in sub-Saharan African population.
Data exclusions	Exclusions was based on standard criteria such as genotype quality (sample missingness and SNP missingness, deviation from Hardy-Weinberg equilibrium, minor allele frequency) for SNPs. Similarly, at the participants level, individuals that were potential duplicates in genetic data, showed inconsistencies between recored and genetic sex or had high missingness were excluded. In additions participants showing extremely high or low lipid levels were also excluded.
Replication	We used a meta-analysis with 4 other African cohorts (UGR, AADM, DDS, DCC) for replicating the signals detected in the Stage 1 of the study. In addition we used data from the GLGC cohort (Grahams et al. 2021) to replicate the novel signals. The replication with respect to GWAS does not involve conducting the same experiment, but rather observing whether the same signals are seen in independent cohorts. We observed replication of many of the previously detected signals in our data. Moreover, some of the signals detected in our data were observed in some of the other cohorts.
Randomization	This study analyses 4 continuous traits and did not allocate any samples to groups therefore randomization is not relevant to our study design.
Blinding	The study does not involve any treatment or sharing of information with participants therefore blinding was relevant to our study design.

Reporting for specific materials, systems and methods

We require information from authors about some types of materials, experimental systems and methods used in many studies. Here, indicate whether each material, system or method listed is relevant to your study. If you are not sure if a list item applies to your research, read the appropriate section before selecting a response.

Materials & experimental systems

n/a	Involved in the study	
x		Antibodies
x		Eukaryotic cell lines
x		Palaeontology and archaeology
x		Animals and other organisms
	x	Human research participants
x		Clinical data
x		Dual use research of concern

Methods

n/a	Involved in the study	
x		ChIP-seq
x		Flow cytometry
x		MRI-based neuroimaging

Human research participants

Policy information about studies involving human research participants

Population characteristics	The participants are population cross-section based with no intended enrichment of any trait/diseases. Most of the participants were in the age range of 40-60 and included both males and females. In addition to self-reported age, sex, ethnolinguistic identity of the participants, whole blood was collected. Population characteristics including covariates are provided in Supplementary Data and Supplementary Information.
Recruitment	A total of 12,000 volunteers included in this study were sampled across six study sites -Agincourt, Dikgale and Soweto in Mpumalanga, Limpopo and Gauteng provinces of South Africa, respectively, Nanoro in Burkina Faso, Novrongo in Ghana and Nairobi in Kenya under the Africa-Wits-INDEPTH partnership for genomic studies (AWI-Gen) project as part of the Human Heredity and Health In Africa (H3Africa) Consortium. The participants were recruited from general population without any specific selection criteria other than a specific age range. Therefore, the results presented are relevant to older-adults.
Ethics oversight	This study was approved by the Human Research Ethics Committee (Medical) of the University of the Witwatersrand (Wits) (protocol number M121029), and renewed in 2017 (protocol number M170880). In addition, research at the Dikgale Study Centre was approved by the Medunsa Research and Ethics Committee of the University of Limpopo (MREC/HS/195/2014:CR). Community engagement preceded sample collection and all participants provided broad consent for medical and population genetic studies.

Note that full information on the approval of the study protocol must also be provided in the manuscript.
